# Echocardiographic strain imaging and progression of atrial fibrillation in low-risk individuals

**DOI:** 10.1016/j.ijcha.2026.101892

**Published:** 2026-02-19

**Authors:** Amelie H. Ohlrogge, Ferdinand Seum, Nick L. van Vreeswijk, Dora Csengeri, Christoph Sinning, Dominik Linz, Michiel Rienstra, Renate B. Schnabel

**Affiliations:** a Department of Cardiology, University Heart and Vascular Center Hamburg, Germany; b German Center for Cardiovascular Research (DZHK), Partner Site Hamburg/Kiel/Lübeck, Hamburg, Germany; c Department of Cardiology, University of Groningen, University Medical Center Groningen, Groningen, the Netherlands; d Department of Biomedical Sciences, Faculty of Health and Medical Sciences, University of Copenhagen, Copenhagen, Denmark; e Department of Cardiology, Cardiovascular Research Institute Maastricht (CARIM), Maastricht University Medical Centre, Maastricht, the Netherlands

**Keywords:** Atrial fibrillation, Progression, Strain, Left Atrium, Left Ventricle

## Abstract

**Background and Aims:**

Left atrial (LA) and left ventricular (LV) strain are sensitive parameters characterizing left ventricular diastolic dysfunction and atrial remodeling. We aimed to assess the association of LA and LV strain and clinical AF progression in two pooled European prospective clinical cohorts.

**Methods:**

Individuals were examined at baseline, with LV global longitudinal strain (GLS), LA reservoir strain (LASr), LA conduit strain (LAScd) and LA contraction strain (LASct) measured by speckle-tracking analysis in transthoracic echocardiography. AF progression was defined as a change from paroxysmal to persistent or persistent to permanent AF. Binary logistic regression was performed to assess the association of baseline strain parameters with AF progression.

**Results:**

Of 316 individuals, 29 (9.2%) had AF progression over a median follow-up of 12 months. Restricted cubic spline modelling suggested non-linear associations between GLS and LASr with AF progression. For LASr only the unadjusted spline model reached statistical significance (p = 0.0498), whereas the adjusted spline model did not (p = 0.074). For GLS, the spline fit indicated a U-shaped curve with a slight trend towards non-linearity (p = 0.072 unadjusted; p = 0.13 adjusted). In unadjusted logistic regression, LASr was significantly associated with AF progression (odds ratio (OR) per standard deviation 0.65, 95% CI 0.42–0.99; p = 0.047).

**Conclusions:**

Our data suggest a potential non-linear association for GLS and LASr as a marker of diastolic function with higher predicted probability of AF progression at lower strain values. However, the test for non-linearity did not reach statistical significance after adjustment.

## Graphical abstract

**Figure:**
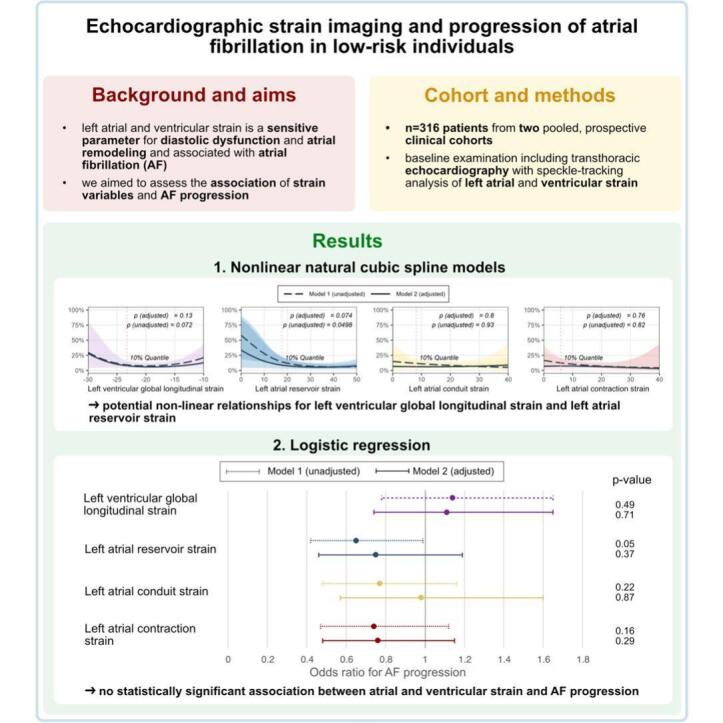

## 1 Introduction

Atrial fibrillation (AF) often is a progressive disease [1]. Higher AF burden has been related to adverse outcomes [2]. As a proxy for AF burden, patients are classified into categories according to the temporal pattern of occurrence. The 2024 ESC guidelines propose a classification into paroxysmal, persistent and permanent AF [3]. During the natural history of AF, the frequency and duration of AF episodes commonly progress. The concept “AF begets AF” reflects the vicious circle, in which AF leads to increasing atrial remodeling, which then again leads to more AF. The progression rate depends largely on the examined population and varies from 0 to more than 10% per year [4], [5]. The progression of AF is associated with an increase of AF-associated complications and adverse events [6], [7], [8] and a decrease in the quality of life [9].

Several risk factors for AF progression have been identified. Clinical variables such as hypertension, age, heart failure among others identify patients at an increased risk of AF progression [6], [10], AF incidence in disease cohorts [11], [12], AF recurrence after cardioversion or ablation [13], [14] or postoperative AF [15]. Beyond these clinical risk factors a variety of markers of atrial cardiomyopathy (AtCM) are associated with AF incidence and progression [16], [17]. These include left atrial (LA) dilatation [6], [9], [18], [19], [20], [21], [22], [23], alterations in the electrocardiogram [24] and biomarkers, in particular N-terminal proBNP [22], [25].

LA and left ventricular (LV) strain are sensitive imaging markers for LV diastolic dysfunction [26] and AtCM [27]. This mechanical dysfunction, which is associated with comorbidities [28], may be impaired before structural changes, such as atrial enlargement, occur [29], [30]. Strain can be measured from cardiac magnetic resonance imaging (MRI), but also from transthoracic echocardiograms. It can be divided according to atrial phase: LA reservoir (LASr), LA conduit (LAScd) and LA contractile strain (LASct). Impaired LA strain values are found in patients with AF and are associated with atrial fibrosis [31]. LA strain and strain rate are associated with LA wall fibrosis on cardiac MRI and AF burden [32]. These changes may precede structural changes [29], and have been associated with incident AF, even in the general population in individuals with a normal sized LA [33], [34]. Reduced LA strain was further associated with incident AF in specific populations, such as patients with a history of ischemic stroke, heart failure, myocardial infarction or hypertension or older patients [35], [36], [37], [38], [39], [40]. Abnormal LV global longitudinal strain (GLS) predicted incident AF in 675 individuals from the population-based Northern Manhattan Study (NOMAS)[41]. GLS was impaired in individuals with AF compared to controls in sinus rhythm [42]. LASr was lower in individuals with persistent AF compared to paroxysmal AF and normalised after restoration of sinus rhythm by catheter ablation [43]. In addition, LA strain has been shown to predict AF recurrence [44]. A Korean study demonstrated a strong association of LA global strain with AF progression [45]. In 196 patients with AF, GLS was a strong predictor of future cardiovascular events [46].

We hypothesized that atrial and ventricular strain measured by two-dimensional speckle-tracking on echocardiography may be associated with AF progression. We tested this hypothesis in two pooled European prospective clinical cohorts.

## 2 Methods

### 2.1 AF-RISK cohort

The *identification of a risk profile to guide atrial fibrillation therapy* (AF-RISK) study was a Dutch prospective, observational multicenter cohort study conducted at the University Medical Centre Groningen and the Maastricht University Medical Centre + between May 2011 and March 2016. It was designed to assess AF progression. Patients ≥ 18 years of age with recent-onset paroxysmal AF or those with a short history of persistent AF with a preferred rhythm control strategy were included. All patients were in sinus rhythm at baseline. Exclusion criteria included a history of heart failure of ≥ 3 years, severe valvular disease, contraindication for oral anticoagulation, acute coronary syndrome within the past months or postoperative AF.

### 2.2 Data collection in the AF-RISK cohort

Baseline data was collected including demographic characteristics, medical history, risk factors, medication and AF symptoms. Patients underwent physical examination, ECG and an exercise test at baseline, and underwent follow-up examinations at 1, 3, 6, 9 and 12 months. For ascertainment of AF and AF burden a 24-hour Holter monitoring was performed at baseline and 6 months, and a 48-hour Holter monitoring and 12 months. AF burden was the time in AF on Holter monitor divided by the total monitoring duration. Additionally, a 2-week event recorder (Vitaphone 100IR, Vitagroup, Mannheim, Germany) was handed out to the patients a baseline and 12 months. A two-dimensional transthoracic echocardiogram was performed at baseline. The endocardial surface was manually traced for speckle tracking analyses of atrial strain using EchoPAC BT12 software (GE HealthCare, Chicago, United States). The additional tracing was generated automatically and checked for accuracy manually. A more detailed cohort description has previously been published elsewhere [22].

### 2.3 AFHRI cohort

The *Atrial Fibrillation in High-Risk Individuals* (AFHRI) study is an ongoing, prospective, monocentric cohort study designed to improve AF risk prediction in patients free of AF at baseline or prevalent AF at risk of disease recurrence. We included patients who were 18 years or older with AF or at an increased risk of developing AF. Individuals who did not have sufficient knowledge of the German language to understand the informed consent forms and to participate in the interview were excluded. In addition, individuals in emergency situations or with acute myocardial infarction were excluded. Participation in the study was voluntary. Written informed consent was obtained from all participants.

### 2.4 Data collection in the AFHRI cohort

Baseline data was collected by an interview using a detailed questionnaire, including information on pre-existing conditions, medication, family history, lifestyle, and cardiovascular risk factors. Baseline information was supplemented by a review of the electronic medical record. Blood was withdrawn and stored at −80 °C. For the determination of AF all electrocardiograms available in the electronic medical record were analyzed by two experienced investigators. In the case of discrepancies in the diagnosis of AF a third cardiologist or electrophysiologist was consulted. Two-dimensional speckle-tacking strain analyses were retrospectively performed by experienced investigators from routine or study transthoracic echocardiograms using TOMTEC Ultrasound Workspace (TOMTEC, Unterschleissheim, Germany) using the AutoStrain function with manual corrections.

The conduct of both studies was approved by the Local Ethics Committee.

### 2.5 Definition of atrial fibrillation progression

For both cohorts AF progression was defined as a change from paroxysmal (≤7 days of continuous AF) to persistent (>7 days of continuous AF), or persistent to permanent AF (AF accepted by patients and physician, no rhythm control pursued). In the AF RISK cohort AF progression was further defined as a doubling of AF burden after 12 months compared to baseline (minimum AF burden 10%) in patients with paroxysmal AF.

### 2.6 Data analysis

Binary logistic regression was performed to assess the association of baseline left atrial strain parameters, LASr, LAScd, and LASct and GLS with atrial fibrillation (AF) progression. Two models were evaluated: Model 1 (unadjusted) and Model 2 (adjusted for age, male sex, body mass index and hypertension). Nonlinear associations were explored via restricted cubic splines with a single knot at the 10th percentile, with likelihood ratio tests comparing spline to linear fits. Models were fitted by maximum likelihood estimation using the logit link. Odds ratios (OR) were calculated per standard deviation increase in each predictor, relative to the predictor’s median as the reference category. ORs were visualized across the strain distribution. Continuous and categorical variables were summarized descriptively. We verified the independence of observations and confirmed that multicollinearity was not present with the variance inflation factor. Statistical significance was defined as a α-level of 0.05. All analyses were performed in R version 4.4.1 using RStudio 2025.05.1 (Posit Software).

## 3 Results

### 3.1 Baseline characteristics

316 individuals were available for analysis. 62.1% were male, with a median age of 59.6 years. A total of 29 individuals had AF progression after a median follow-up of 12 months, resulting in an overall 9.2% AF progression and a 7.1% yearly AF progression. Those with AF progression were significantly older (65.5 vs. 59.4 years, p = 0.015) than individuals in the group without AF progression, without significant sex differences (p = 0.99). Body mass index (BMI), systolic and diastolic blood pressure did not differ significantly between groups. Hypertension, sleep apnoea, diabetes and current smoking were prevalent at baseline in 44.6%, 4.7%, 8.5% and 14.6%, respectively. Hypertension was significantly more common in the AF progression group (65.5% vs. 42.5%, p = 0.018), whereas the prevalence of sleep apnoea, diabetes and current smoking did not differ significantly between groups. Median GLS in the total cohort was −19.8% (Interquartile range [IQR] −21.6–-17.2%), within the normal range reported in previous literature, without significant differences between cohorts [47]. Left atrial strain parameters were available in 297 individuals, including 27 with AF progression. In this cohort median LASr was 32.7% (IQR 24.4–41.5%), median LAScd 17.6% (11.3–23.7%), median LASct 14.3% (10.6–19.1%), again without significant differences between groups. These strain values are lower than reported normal values for healthy individuals [48], and in alignment with reported values for individuals with AF [49].

**Fig. 1:**
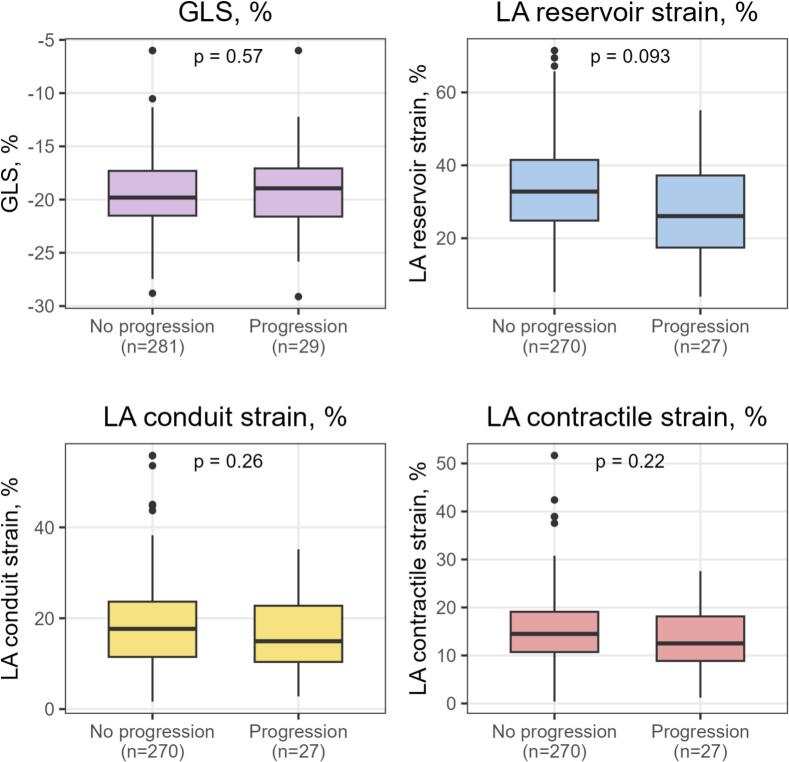
Boxplots of GLS (lavender) and LASr (blue), LAScd (yellow), and LASct (red), by AF progression status. Boxes indicate interquartile range, horizontal lines the median, and whiskers the minimum and maximum values. Abbreviations: GLS – global longitudinal strain, LA – left atrial. (For interpretation of the references to colour in this figure legend, the reader is referred to the web version of this article.)

Participants with AF progression (n = 29) showed numerically lower left atrial strain values compared with those without AF progression (n = 287), whereas GLS values were largely comparable between groups. The largest between-group difference was observed for left atrial reservoir strain (26.1% [17.4–37.2] vs. 32.8% [24.8–41.5]; p = 0.093). Left atrial conduit strain (14.9% [10.4–22.8] vs. 17.7% [11.5–23.6]; p = 0.26) and left atrial contractile strain (12.5% [8.9–18.1] vs. 14.5% [10.7–19.1]; p = 0.22) were also numerically lower in participants with AF progression, whereas GLS did not differ materially between groups (−18.9% [−21.6 to − 17.1] vs. − 19.8% [−21.5 to − 17.3]; p = 0.57). Baseline use of rate-control therapy, RAAS inhibitors/diuretics, and anticoagulation was numerically more frequent among participants with AF progression, whereas rhythm-control therapy was similarly distributed between groups; however, no statistically significant differences in medication use were observed (Table 1**,** Fig. 1).

**Table 1:** Baseline characteristics. For categorical variables, N are provided, percentages are shown in brackets. For continuous variables the median is shown, with the 25th and 75th percentile displayed in brackets). Comparisons between groups were done by Mann–Whitney *U* test. * p-value for difference between AF progress and no AF progress. Left atrial strain (LASr, LAScd and LASct) were available in n = 297 individuals, 27 of these had AF progression. Abbreviations: AF − atrial fibrillation, LAScd – left atrial conduit strain, LASct − left atrial contractile strain, LASr – left atrial reservoir strain, GLS, Global longitudinal strain, RAAS – Renin-angiotensin-aldosterone system.

Variable	Total cohortn = 316	AF progression n = 29	No AF progression n = 287	p-value
Clinical characteristics				
Men	196/316 (62.0%)	18/29 (62.1%)	178/287 (62.0%)	0.99
Age, years	59.6 (50.3–66.6)	65.5 (53.7–69.5)	59.4 (50.0–65.8)	0.015
Body mass index, kg/m^2^	26.6 (24.3–29.8)	26.8 (24.4–29.7)	26.6 (24.3–29.9)	0.63
Systolic blood pressure, mm Hg	130 (120–140)	130 (119–140)	130 (120–140)	0.81
Diastolic blood pressure, mm Hg	80 (70–80)	79 (70–80)	80 (70–80)	0.32

Pre-existing conditions at baseline				
Hypertension	141 (44.6%)	19 (65.5%)	122 (42.5%)	0.018
Sleep apnoea	15 (4.7%)	3 (10.3%)	12 (4.2%)	0.14
Diabetes	27 (8.5%)	4 (13.8%)	23 (8.0%)	0.29
Current smoking	46/316 (14.6%)	6/29 (20.7%)	40/287 (13.9%)	0.32

Echocardiographic strain parameters				
GLS, %	−19.8 (−21.6–-17.2)	−18.9 (−21.6–-17.1)	−19.8 (−21.5–-17.3)	0.57
LASr, %	32.7 (24.4–41.5)	26.1 (17.4–37.2)	32.8 (24.8–41.5)	0.093
LAScd, %	17.6 (11.3–23.7)	14.9 (10.4–22.8)	17.7 (11.5–23.6)	0.26
LASct, %	14.3 (10.6–19.1)	12.5 (8.9–18.1)	14.5 (10.7–19.1)	0.22

Medication				
Rate-control medication, %	71/316 (22.5%)	10/29 (34.5%)	61/287 (21.3%)	0.10
Rhythm-control medication, %	46/316 (14.6%)	4/29 (13.8%)	42/287 (14.6%)	0.90
RAAS inhibitors/diuretics, %	88/316 (27.8%)	12/29 (41.4%)	76/287 (26.5%)	0.088
Anticoagulation, %	203/316 (64.2%)	23/29 (79.3%)	180/287 (62.7%)	0.076

Echocardiographic system				
TOMTEC Ultrasound Workspace	34/316 (10.8%)	3/29 (10.3%)	31/287 (10.8%)	0.94
GE EchoPAC BT12	282/316 (89.2%)	26/29 (89.7%)	256/287 (89.2%)	0.94

### 3.2 Nonlinear natural cubic spline models

**Fig. 2:**
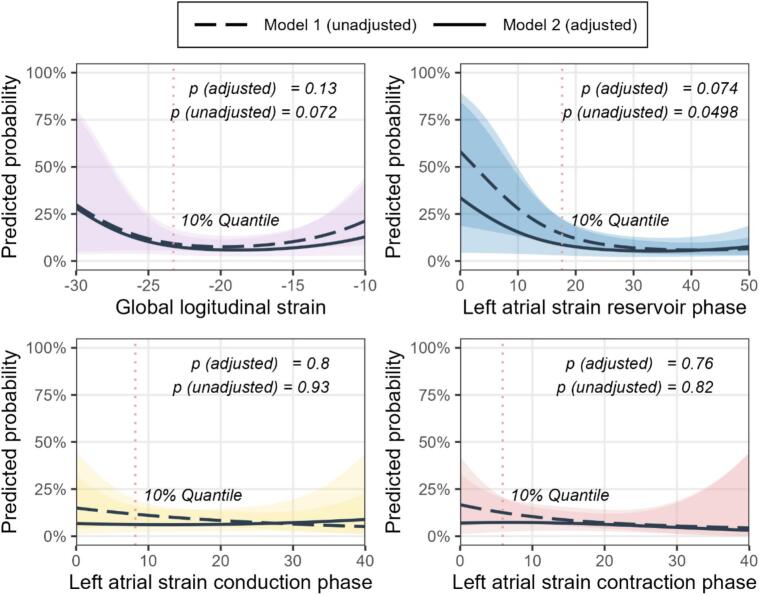
Restricted cubic spline plots of GLS (purple), left atrial reservoir (blue), conduit (red), and contractile (yellow) strain with predicted probability of AF progression on the y-axis, calculated for a reference profile with all covariates fixed at their median values (age, sex, BMI, hypertension, current smoking, echo system, rate-control medication, rhythm-control medication, RAAS/diuretics, anticoagulation). Splines use a single knot at the 10th percentile. P-values indicate the significance of the spline fit compared with the linear model, tested by likelihood ratio test. (For interpretation of the references to colour in this figure legend, the reader is referred to the web version of this article.)

Restricted cubic spline modelling suggested non-linear associations between GLS and LASr with the predicted probability of AF progression in a reference profile of a male participant without hypertension, with median age and median BMI. For GLS, the likelihood ratio test indicated a trend towards non-linearity in the unadjusted model (p = 0.072), which was attenuated after adjustment (p = 0.131). For LASr, evidence of non-linearity was observed in the unadjusted(p = 0.0498), while the adjusted model showed a similar, though non-significant, trend (p = 0.074) (Fig. 2Although increased variability was observed at the extremes of the strain distribution, lower strain values were consistently associated with higher predicted probabilities of AF progression. No evidence for non-linearity was found for LAScd (p = 0.93 unadjusted; p = 0.8 adjusted) or LASct (p = 0.82 unadjusted; p = 0.76 adjusted).

### 3.3 Logistic regression analysis

In unadjusted models (Model 1), LASr showed a borderline association with AF progression (OR per SD 0.65, 95% CI 0.42–0.99; p = 0.047) (Table 2). GLS (OR 1.14, 95% CI 0.78–1.65; p = 0.49), LAScd (OR 0.77, 95% CI 0.48–1.16; p = 0.22), and LASct (OR 0.74, 95% CI 0.47–1.12; p = 0.16) were not statistically significant. In adjusted analyses (Model 2; adjusted for age, male sex, BMI, hypertension, current smoking, echo system, rate-control medication, rhythm-control medication, RAAS inhibitors/diuretics, and anticoagulation), the association of LASr was markedly attenuated (OR 0.86, 95% CI 0.51–1.43; p = 0.55), and none of the strain parameters reached statistical significance. Similar null associations were observed for GLS (OR 1.04, 95% CI 0.70–1.54; p = 0.85), LAScd (OR 1.07, 95% CI 0.62–1.84; p = 0.80) and LASct (OR 0.84, 95% CI 0.52–1.36; p = 0.48). Overall, the results do not support strong effects of strain measures on AF progression in this cohort.

**Table 2:** Odds ratios (OR) per standard deviation (SD) increase in GLS and left atrial strain parameters (LASr, LAScd, LASct) for prediction of AF progression. Model 1 = unadjusted; Model 2 = adjusted for age, male sex, BMI, hypertension, current smoking, echo system, rate-control medication, rhythm-control medication, RAAS inhibitors/diuretics, anticoagulation.

	Model 1		Model 2	
	OR (95% CI)	p-value	OR (95% CI)	p-value
GLS, per SD	1.14 (0.78, 1.65)	0.49	1.04 (0.70, 1.54)	0.85
LASr, per SD	0.65 (0.42, 0.99)	0.047	0.86 (0.51, 1.43)	0.55
LAScd, per SD	0.77 (0.48, 1.16)	0.22	1.07 (0.62, 1.84)	0.80
LASct, per SD	0.74 (0.47, 1.12)	0.16	0.84 (0.52, 1.36)	0.48

## 4 Discussion

In this combined analysis of two prospective, European cohorts LASr was significantly associated with AF progression in an unadjusted logistic regression model, whereas this association did not persist after multivariable adjustment. Neither LA nor LV strain parameters were independently associated with AF progression in adjusted logistic regression models. This attenuation is likely explained by confounding from baseline clinical risk factors, particularly age and hypertension, which were more prevalent among participants with AF progression.

Spline analyses suggested that GLS and LASr may have non-linear relationships with AF progression. No non-linear patterns were observed for LAScd and LASct. For GLS the risk of AF progression followed a U-shaped pattern, with higher predicted probabilities of AF progression at low and high strain values, alongside increased variability at the extremes of the strain distribution. Whereas higher GLS (i.e. less negative values) reflects systolic dysfunction and thus falls into typical pathological cut-offs, unusually low GLS may represent hyperdynamic ventricles, and may be related to underlying abnormalities in the circulatory or metabolic system, which in turn might be associated with an increased risk of AF progression. Similar U-shaped associations between GLS and adverse cardiovascular outcomes, including hospitalization and mortality, have been reported previously [50]. Likewise, increased left ventricular ejection fraction has been associated with an increased incidence of a combined endpoint (all-cause death, non-fatal myocardial infarction, and heart failure) in both women and men and an increased mortality in women [51], [52]. These findings highlight the importance of considering non-linear modelling in strain analyses to better reflect the complex underlying biological mechanisms.

Atrial strain is an established predictor of AF recurrence, both after electrical cardioversion and catheter ablation. LASr and LASct were independent predictors of atrial tachyarrhythmia recurrence after catheter ablation in n = 678 individuals [53]. LASr predicted both 1-year and 2-year AF recurrence in n = 380 individuals undergoing catheter ablation [54]. Global peak atrial longitudinal strain predicted AF recurrence after electrical cardioversion in individuals with persistent and long-standing persistent non-valvular AF [55]. Among individuals undergoing cardioversion, LASr independently predicted the recurrence of AF, whereas LA strain and GLS improved in those without AF recurrence [56]. In individuals with atrial fibrillation undergoing electrical cardioversion, global atrial strain recovered in sinus rhythm in only 33% of participants during a follow-up of 3 months [57].

Though numerous studies have demonstrated an association of left atrial and ventricular strain and AF incidence or recurrence, previous evidence on the association between left atrial strain and the progression of AF is limited. A Korean cohort with 313 participants showed an association of left atrial strain and AF progression [45]. In 417 patients from the reappraisal of AF: Interaction Between HyperCoagulability, Electrical Remodelling, and Vascular Destabilisation in the Progression of AF (RACE V) cohort, LA contractile and reservoir function, though not LA conduit function or LV strain were significantly lower in those with AF progression. Impaired LA contractile function was a significant predictor for AF progression in multivariable analysis [58]. These few, positive studies indicate that our neutral findings might be related to a lack of statistical power rather than the true absence of an association, though publication bias needs to be considered. Evidence on the predictors of AF progression does not only provide pathophysiological insights into the mechanisms behind progressive AtCM, but may be of clinical utility in order to identify high-risk individuals for intensified surveillance and secondary prevention efforts. Further research is needed to assess the relationship between LA and LV strain as markers of atrial remodeling and diastolic dysfunction and the progression of AF and AtCM.

### 4.1 Strengths and limitations

A major strength of our analysis is the comparatively large number of well-phenotyped individuals in this two-center analysis of prospective European clinical cohorts. However, due to distinct differences in study design the data could not be merged for all participants. This fact reduced our sample size for some analyses and thus statistical power. Based on the effect size reported by Yoon et al.[45], statistical power was conservatively estimated for analyses of left atrial strain, which had fewer available observations and AF progression events than analyses of GLS. In the left atrial strain analyses (n = 297, with 27 AF progression events),the calculated power was approximately 65%, corresponding to a type II error risk of about 35%. Thus, it remains possible that a true association between LA strain and AF progression was not detected in our analysis due to the limited number of events. Although analyses were adjusted for vendor-specific software platforms, differences in absolute strain values between imaging systems cannot be fully excluded, as demonstrated by Farsalinos et al.[59] As common in studies in patients without implanted cardiac devices, we do not have an exact quantification of AF burden. The clinical definition of AF progression may have led to misclassification of our outcome of AF progression.

## 5 Conclusion

In this combined analysis of two prospective, European cohorts at low risk of AF progression neither left atrial nor left ventricular strain parameters were strongly associated with AF progression. Spline analyses indicated potential non-linear relationships, in particular for LASr and GLS.

## CRediT authorship contribution statement

**Amelie H. Ohlrogge:** Writing – review & editing, Writing – original draft, Visualization, Validation, Project administration, Methodology, Data curation, Conceptualization. **Ferdinand Seum:** Writing – review & editing, Writing – original draft, Visualization, Validation, Software, Methodology, Formal analysis, Data curation, Conceptualization. **Nick L. van Vreeswijk:** Writing – review & editing, Resources, Project administration, Data curation. **Dora Csengeri:** Writing – review & editing, Project administration, Data curation. **Christoph Sinning:** Writing – review & editing, Supervision, Project administration, Data curation. **Dominik Linz:** Writing – review & editing, Project administration, Data curation. **Michiel Rienstra:** Writing – review & editing, Supervision, Resources, Project administration, Methodology, Funding acquisition, Formal analysis, Data curation, Conceptualization. **Renate B. Schnabel:** Writing – review & editing, Writing – original draft, Supervision, Project administration, Investigation, Funding acquisition, Formal analysis, Data curation, Conceptualization.

## Funding

The GGAF (Groningen Genetics of Atrial Fibrillation) is supported by funding to the 5 sources that form GGAF. The AF RISK study is supported by the Netherlands Heart Foundation (grant NHS2010B233), and the Center for Translational Molecular Medicine. Both the Young-AF and Biomarker-AF studies are supported by funding from the University Medical Center Groningen. The GIPS-III trial was supported by grant 95103007 from ZonMw, the Netherlands Organization for Health Research and Development. The PREVEND study is supported by the Dutch Kidney Foundation (grant E0.13) and the Netherlands Heart Foundation (grant NHS2010B280).

AO is supported by the German Heart Foundation/German Foundation of Heart Research. DC received a research grant from Wolfgang Seefried Project funding of the German Heart Foundation. MR has received the following funding: Unrestricted research grant from the Dutch Heart Foundation which is conducted in collaboration with and supported by the Dutch CardioVascular Alliance, 01-002-2022-0118 EmbRACE, an unrestricted research grant from ZonMW and the Dutch Heart Foundation; DECISION project 848090001, an unrestricted research grants from the Netherlands Cardiovascular Research Initiative: an initiative with support of the Dutch Heart Foundation; RACE V (CVON 2014–9), RED-CVD (CVON2017-11). Unrestricted research grant from Top Sector Life Sciences & Health to the Dutch Heart Foundation (PPP Allowance; CVON-AI (2018B017)), an unrestricted research grant from the European Union’s Horizon 2020 research and innovation programme under grant agreement; EHRA-PATHS (945260).

RBS has received funding from the European Research Council (ERC) under the European Union’s Horizon 2020 research and innovation programme under the grant agreement No 648131, from the European Union’s Horizon 2020 research and innovation programme under the grant agreement No 847,770 (AFFECT-EU) and German Center for Cardiovascular Research (DZHK e.V.) (81Z1710103 and 81Z0710114); German Ministry of Research and Education (BMBF 01ZX1408A) and ERACoSysMed3 (031 L0239). Wolfgang Seefried project funding German Heart Foundation.

All authors take responsibility for all aspects of the reliability and freedom from bias of the data presented and their discussed interpretation.

## Declaration of competing interest

The authors declare the following financial interests/personal relationships which may be considered as potential competing interests: NLvV received a speaker fee from Daiichi-Sankyo. On behalf of DL, the Maastricht University Medical Center received consulting fees/honoraria by Acesion, Bayer, Biotronik, Boston Scientific, CardioFocus, iRhythm, Johnson & Johnson MedTech, Medtronic, Novartis, NovoNordisk. MR received Consultancy fees from Bayer (OCEANIC-AF national PI), InCarda Therapeutics (RESTORE-SR national PI), Novartis to the institution and a speaker fee from Daiichi-Sankyo, Pfizer to the institution. RBS has received lecture fees and advisory board fees from BMS/Pfizer outside this work.
